# Development, implementation, and evaluation of interprofessional events on climate change in health professions curricula

**DOI:** 10.3389/fmed.2025.1736224

**Published:** 2026-01-06

**Authors:** Kin Ly, Alethea Cariddi, Michelle Cote, Kris Hall

**Affiliations:** 1 Department of Biomedical Sciences, University of New England College of Osteopathic Medicine, Portland, ME, United States; 2 Office of Sustainability, University of New England, Biddeford, ME, United States; 3 Center to Advance Interprofessional Education and Practice, University of New England, Portland, ME, United States

**Keywords:** climate change, health professions education, human health, interprofessional education, planetary health

## Abstract

The threat of climate change and its negative effects on human and planetary health is at the forefront of health organizations around the world. Advocacy to integrate climate change content into health professions education is supported by evidence found in academic journals and promoted widely by academic health organizations. While some health professions schools have accomplished this, many have yet to integrate climate change into their curricula. In 2024, the University of New England College of Osteopathic Medicine collaborated with the university’s Center to Advance Interprofessional Education and Practice and its Planetary Health Council to co-create two interprofessional education events. These events prioritized the introduction of medical and other health professions students to the impacts of the climate crisis on human and environmental health through innovative co-curricular programming that brought together students from multiple disciplines. This descriptive study analyzes post-event surveys and qualitative data to examine event outcomes and recommendations to guide future event planning.

## Introduction

According to the World Health Organization (WHO), climate change is a fundamental threat to human health (1). Climate change causes extreme weather events that are becoming more frequent and adversely affecting the health outcomes of the world population. As reported by the United States (US) Centers for Disease Control and Prevention (CDC), climate change impacts human health in various ways (2) such as: (1) worsening air pollution, leading to increased incidents of asthma and premature deaths (3); (2) threatening food quality, production, and distributions systems, leading to global food insecurity (4); (3) changing weather conditions, leading to increased food and waterborne diarrheal disease (5); and (4) increasing extreme weather events and disasters, causing mental health issues in those with no past medical history and exacerbating mental health conditions in people with previous histories (6).

Climate change causes adverse effects on human health, but the medical industry itself is a major contributor to the climate crisis (7). According to Downey (8), one of the significant causes of climate change is the healthcare industry, which produces enormous amounts of waste. The climate crisis is further accelerated by the atmospheric emissions caused by the manufacturing, transportation, and disposal of hazardous waste and plastics. In 2019, the Organization for Economic Co-operation and Development (OECD) reported on the pharmaceutical waste problem that has catastrophic consequences to the environment (9). While pharmaceuticals are beneficial to humans and animals, residues from drugs such as hormones, antidepressants, and antibiotics are discharged into surface water and groundwater globally (9). Research has shown that:

Oral contraceptives have caused the feminization of fish and amphibians; psychiatric drugs, such as fluoxetine, alter fish behavior making them less risk-averse and vulnerable to predators; and the over-use and discharge of antibiotics to water bodies exacerbates the problem of antimicrobial resistance – declared by the World Health Organization as an urgent, global health crisis that is projected to cause more deaths globally than cancer by 2050 (9).

In recent years, more attention to climate change in medical education has occurred with publications urging medical schools (10) and other health professions schools (11) to include climate change content in their school curricula to prepare future healthcare professionals (12) with a better understanding of the impacts climate change has on public and individual health. Some medical schools such as Harvard Medical School (13) and Stanford School of Medicine (14) have integrated climate change into their schools’ curricula while other schools have yet to accomplish this. According to the results of the Curriculum SCOPE Survey conducted by the Association of American Medical Colleges (AAMC) regarding the impacts of climate change on health curricula at US medical schools, 121 out of 166 (73%) allopathic and osteopathic medical schools surveyed included health effects of climate change in their 2023–2024 curricula (15).

The Planetary Health Report Card (PHRC) for the University of New England College of Osteopathic Medicine (UNE COM) in 2023 achieved an overall grade of C, C- in 2024, and C in 2025 (16–18). Due to these disappointing results, and the increasing urgency to address climate change and its effects on human and planetary health with future healthcare providers, a faculty member from UNE COM collaborated with the UNE Center to Advance Interprofessional Education and Practice (CAIEP), and the UNE Planetary Health Council (PHC) to create an interprofessional education (IPE) event. A second IPE event, later that year hosted by CAIEP and PHC, was promoted broadly to all health professions at the university and was well attended. These ongoing IPE events, known as CAIEP Knowledge Exchanges, highlighted the public health impacts caused by environmental pollutants and climate change. They were, “*The Effects of Pharmaceutical Waste on Human and Planetary Health*” and “*Thriving Waters: Navigating the Health and Business of Aquaculture*” (19).

The purpose of this study was to determine the participants’ program affiliation, assess the outcomes of these climate-and pollutant-focused IPE events, and explore ways in which they can be improved, based on participant feedback.

## Methods

This post-event descriptive study used a post-event survey based on the study’s objectives. The Office of Research Integrity at UNE did not require IRB review and approval because this study is not research involving human subjects as defined by 45 CFR 46.102.

Study participants included administrative staff, faculty members, and students from UNE who attended the two IPE events in 2024 via Zoom or livestream. At each IPE event, after panelists presented their content, discussions were held in breakout rooms or through a Q&A session; both were moderated by a faculty or student facilitator. Faculty and student facilitators were not trained prior to the two IPE events. Discussion prompt(s) were provided. Participants were shown a QR code at the end of each IPE event that led to an online post-event survey through Google Forms.

The post-event survey used a series of closed and open-ended questions. The closed-ended questions were single-select multiple-choice questions and multiple-response questions. Data collected from the participants identified their program of study or status as an administrator, faculty member, or staff, and their qualitative feedback on what was successful about the event and what could be changed was analyzed.

Collected data was exported to Excel spreadsheet and deidentified. Open-ended responses about the event successes and submitted changes were reviewed, coded, and themes were identified by the primary author (KL). ChatGPT was then used to code and identify themes, which were reviewed by KL, and found to align with the codes and themes previously identified.

## Results

Attendance at the first IPE event, “*The Effects of Pharmaceutical Waste on Human and Planetary Health*,” held on February 28, 2024 was larger than the second event, “*Thriving Waters: Navigating the Health & Business of Aquaculture*,” held on October 23, 2024. There were 224 on Zoom and 3 on livestream with 206 completing the post-event survey for the first IPE event. One post-event survey response was excluded due to lack of identification of the academic program for a total of 205. The response rate was 90.3%. For the second event there were 123 on Zoom and 8 on livestream with 86 completing the post-event survey. Two post-event survey responses were excluded due to lack of identification of the academic program for a total of 84. The response rate for the second event was 64.1%.

Table 1 shows the number of participants for both IPE events and academic programs. Students from the Osteopathic Medicine program were the majority of attendees for both IPE events, but also in attendance were students from various undergraduate and graduate programs.

**TABLE 1:** Composition of attendees for each interprofessional education event.

Interprofessional education events	The Effects of Pharmaceutical Waste on Human and Planetary Health (2/28/24)	Thriving Waters: Navigating the Health & Business of Aquaculture (10/23/24)
**Undergraduate programs**		
Applied exercise science		1
Dental hygiene	14	
Environmental science		1
Health, wellness, and occupational studies	2	
Marine sciences		1
Medical biology	1	
Nursing		1
Public health	1	
**Graduate programs**		
Applied nutrition	2	2
Dental medicine	3	1
Osteopathic medicine	171	65
Pharmacy	4	7
Public health	1	1
**Others**		
Faculty/staff	6	4
Total	205	84

Five themes, frequency, and representative quotes for each theme, based on post-event survey responses, can be found in Tables 2, 3. Descriptions of the themes can be found in the following qualitative responses.

**TABLE 2:** Themes, frequency, and representative quotes for statement, “Please let us know what was successful about today’s event.”

Themes	Frequency	Representative quotes
**Event 1: The Effects of Pharmaceutical Waste on Human and Planetary Health**		
Active and inclusive engagement	46	“I enjoyed the breakout rooms and discussing the topic with other health profession students.”
		“I really like the opportunity to break out into smaller rooms in order to discuss these issues in a smaller group to increase student engagement.”
Rich interdisciplinary learning	45	“It was engaging to speak with other medical professional, to see their viewpoint on the topic.”
		“Hearing from many different perspectives and knowledge-bases (pharmacist, DEA agent, etc.) was very insightful.”
Effective organization and presentation	29	“Very helpful to have the slides available for us to view.”
		“It was well presented - organized and effective flow.”
Expanding awareness of environmental health	55	“I learned a lot about pharmaceutical waste and environmental concerns that I was not aware of previously!”
		“Very informative about a topic that is commonly overlooked.”
Professional relevance and motivation to act	13	“So learning about what our role could be is helpful.”
		“I plan to implement this knowledge into my future practice.”
**Event 2: Thriving Waters: Navigating the Health and Business of Aquaculture**		
Rich interdisciplinary learning	21	“I loved the inclusion of nutrition, as that is a topic that doctors do not often cover in class.”
		“Being able to bring together panelists from different yet meshing industries and disciplines…”
Speaker expertise and diversity	18	“I enjoyed listening to all the speakers and hearing the different perspectives and insights!”
		“The diversity of expertise represented by the panelists.”
rowspan="3"Real-world perspectives	15	“I liked seeing…insight and learning about his experience and history in the aquaculture industry.”
		“I loved hearing from…about his personal perspective as an oyster farmer.”
Organization and flow	8	“The presentation was well laid out and the flow was consistent and easy to follow”
		“Great Talk! Very organized and informative.”
Engagement and discussion	27	“I think it was an engaging topic and I liked the speakers…”
		“Great discussion and great answers to questions asked.”

**TABLE 3:** Themes, frequency, and representative quotes for statement, “Please let us know what you would change about today’s event.”

Themes	Frequency	Representative quotes
**Event 1: The Effects of Pharmaceutical Waste on Human and Planetary Health**		
High overall satisfaction	57	“Nothing.”
		“Nothing! I think the event went very smoothly…”
Breakout room challenges	18	“Optimize breakout rooms - automatically assign rooms to avoid overcrowding…”
		“Each breakout room did not have a facilitator and the rooms were not broken up evenly.”
Scheduling and time constraints	11	“Maybe make it only until 12:45 pm so that we can make it to our next class without rushing.”
		“Tough time during lunch.”
Desire for deeper and more applied content	12	“I would have loved to learn what environmental policies or activism is already in progress…”
		“I would have liked to discuss more of what we could do as individuals to create a change.”
Presentation and platform improvements	5	“A lot of words on the slides, trying to read can distract from what the presenters are saying.”
		“What I would change about today’s event is to not have a lot of people chatting in the chat feature on zoom when the speakers are talking.”
**Event 2: Thriving Waters: Navigating the Health and Business of Aquaculture**		
High overall satisfaction	25	“I wouldn’t change anything.”
		“Nothing! I enjoyed the entire presentation.”
Desire for extended Q&A and better time management	7	“Seems like there were a lot of questions so potentially provide further time for those.”
		“There was so much great information, wish there was more time for more questions at the end.”
Improved facilitation and flow	2	“Encouraging a student moderator to stay involved with the discussion and keep things moving along.”
		“It may be helpful to prompt progress to next questions after a few minutes of discussion on a question.”
Inclusion of visual aids and learning supports	1	“I’m a visual learner, so keeping slides up to go along with the discussion would be helpful.”
More applied and interdisciplinary content	2	“I would have also loved to see public health implications of improperly grown seafood.”
		“A panelist who could have been able to speak to the benefits of these nutritional benefits and aquaculture to medicine…”

Qualitative responses to the first IPE event for what was successful showed that participants: (1) consistently highlighted the interactive breakout rooms, discussions in the Chat, and strong facilitation as key to meaningful participation and comfort in sharing; (2) appreciated the multiple perspectives of the panelists and other healthcare students, which contributed to their learning; (3) were engaged when the technical and structural organization of the event was well prepared; (4) felt the topic of pharmaceutical waste and sustainability was timely, underdiscussed, and impactful; and (5) connected the content to their future healthcare roles and expressed motivation to apply what they learned.

Qualitative responses to what should be changed for the first IPE event identified the following: (1) a strong majority expressed that the session was effective and needed no change; (2) frequent area for improvement was the breakout rooms due to uneven group sizes, lack of facilitators, and unclear discussion flow; (3) the time-slot of the event was difficult since it was held during the lunch break time and the short available time for students to get to their next class; (4) some wanted expanded discussion on real-world implications, policies, or activism around pharmaceutical waste; and (5) greater clarity of the slides and fewer distractions in the Chat.

Qualitative responses to the second IPE event for what was successful showed that participants: (1) appreciated the event’s integration of environmental science, nutrition, and healthcare, which deepened their understanding of human and ecological health; (2) cited the variety of panelists with different backgrounds was key; (3) found the personal experiences from one of the panelists made the IPE event more engaging and grounded in reality; (4) highlighted that the event was well organized and the delivery of the content flowed smoothly; and (5) felt that the Q&A and discussion enriched the IPE event.

Qualitative responses to what should be changed for the second IPE event identified the following: (1) participants expressed satisfaction of the event with no need for changes; (2) the lack of enough time for questions and discussion and the need for more opportunities to engage with the panelists and other students; (3) the need for facilitation training to help the student facilitator better manage the pacing and transitions between topics; (4) having the slides displayed during discussions for visual learners was desired; and (5) a few participants wanted deeper connections between aquaculture, healthcare/public health and their healthcare training.

## Discussion

According to the United Nations, human activity is the main driver of climate change (20). Changes in temperature and weather are linked to climate change, which affects human and planetary health. Developing countries are more vulnerable to the impact of climate change, but people globally feel its consequences, regardless of where they live (20). In order to address the need for future healthcare professionals to know and understand the effects of climate change on public and individual health, UNE COM partnered with the university’s CAIEP and its PHC to co-create two IPE events. These IPE events prioritized the intersection of human and environmental health, the first focused on pharmaceutical waste and the second on aquaculture in the face of climate change.

In 2010, the WHO published, “*Framework for Action on IPE & Collaborative Practice*” (21). According to the framework, “IPE occurs when students from two or more professions learn about, from and with each other to enable effective collaboration and improve health outcomes. Once students understand how to work interprofessionally, they are ready to enter the workplace as a member of the collaborative practice team” (21). For these events, IPE pedagogy was used to foster students’ appreciation for the benefits of collaborative teamwork in addressing highly complicated and challenging public and global health issues. The intentional interactional format of the events and its cross-professional design prepare future healthcare professionals to communicate effectively (22), understand their roles and responsibilities as healthcare professionals (22), effectively collaborate as team members (22), apply ethical and professional standards to ensure safe patient-centered care (23), and promote the role of healthcare professionals as public health advocates.

Center to Advance Interprofessional Education and Practice’s Knowledge Exchanges are well-recognized co-curricular activities at UNE that invite shared learning on contemporary topics that affect the health of individuals, communities, and the planet. Each IPE event incorporates specific Interprofessional Education Collaborative (IPEC) Core Competencies for Interprofessional Collaborative Practice (24) aligned with the selected topic; in this instance values and ethics of teamwork and cross-professional communication were chosen to underscore the relevance of understanding the impacts of climate change as future healthcare professionals. Post-event comments indicated that being exposed to how the climate crisis and environmental toxins affect the world population and also how healthcare will be delivered in the future were valued by the participants.

Studies find that participation in IPE activities advances students’ cross-professional communication skills and bolsters their commitment as team members to improve healthcare delivery (25–27). In this study, participants described overall satisfaction with co-curricular learning, stating that both IPE events provided rich interdisciplinary learning experiences and opportunities to discuss planetary health and climate change and its impacts with other health professions while also learning from the diverse expertise of panelists.

Challenges were also identified in this study. Scheduling is a common barrier to providing interprofessional activities (28) and the timing of these IPE events was no exception. Integration of IPE learning into curriculum is a way to ameliorate this concern or alternatively, designating university-wide time for shared learning could eliminate scheduling issues.

Breakout sessions also posed challenges for participants with some noting unevenness in facilitation skills across groups. IPE facilitation is a complex and demanding role, and it is recommended that faculty and others facilitating events receive additional training and support in IPE facilitation (29). Requiring pre-event facilitation and information technology services training for those volunteering to facilitate breakout rooms is a reasonable solution to improve the student experience. However, given time constraints and the voluntary nature of facilitation it would be difficult to enforce this requirement.

There are limitations to this study. The qualitative methodology of this study offers institution specific and valuable findings for curricular improvements. However, due to its qualitative nature, the results are only applicable to one university and cannot be broadly generalized to other institutions. To address this limitation, future scholarship can include quantitative methodology to assess participants’ responses to such IPE events to provide more generalizable and statistically sound results. Another limitation is that UNE medical students made up the bulk of attendance at the two IPE events, thus breakout rooms were populated by a majority of students from the medical school compared to the other health professions programs. Although participant feedback did not mention the predominance of medical students, it is likely that breakout room conversations may have been dominated by them. Efforts to identify reasons for low attendance by other health professions students are needed to improve the balance and representation of their perspectives and scope of practice in these IPE events.

## Conclusion

The two IPE events were a promising start to providing climate change and planetary health as co-curricular education for the health professions programs at UNE. Utilizing IPE pedagogy to expose students to concepts of planetary health and its intersection with human health and healthcare prepares them with workplace skills such as effective communication and value for working in teams. Exposure to global environmental and social concerns also offers impetus for future healthcare professionals to engage in advocacy and interdisciplinary research that improve planetary and human health. Currently, UNE plans to continue hosting IPE events that foster awareness of the interconnections among climate change, environmental pollutants, and planetary health.

## Data Availability

The original contributions presented in this study are included in this article/supplementary material, further inquiries can be directed to the corresponding authors.
